# The Impact of the PI3K/Akt Signaling Pathway in Anxiety and Working Memory in Young and Middle-Aged PDK1 K465E Knock-In Mice

**DOI:** 10.3389/fnbeh.2020.00061

**Published:** 2020-05-08

**Authors:** Lydia Giménez-Llort, Mikel Santana-Santana, José Ramón Bayascas

**Affiliations:** ^1^Institut de Neurociències, Universitat Autònoma de Barcelona, Barcelona, Spain; ^2^Department of Psychiatry and Forensic Medicine, School of Medicine, Universitat Autònoma de Barcelona, Barcelona, Spain; ^3^Department of Biochemistry and Molecular Biology, School of Medicine, Universitat Autònoma de Barcelona, Barcelona, Spain

**Keywords:** RDoC, PI3K/Akt, signaling pathway, anxiety, cognition, animal model, aging, fine tuning

## Abstract

Dysfunction and dysregulation at the genetic, neural, and behavioral levels point at the fine-tuning of broadly spread networks as critical for a wide array of behaviors and mental processes through the life span. This brain-based evidence, from basic to behavioral neuroscience levels, is leading to a new conceptualization of mental health and disease. Thus, the Research Domain Criteria considers phenotypic differences observed among disorders as explained by variations in the nature and degree of neural circuitry disruptions, under the modulation of several developmental, compensatory, environmental, and epigenetic factors. In this context, we aimed to describe for the first time the *in vivo* behavioral impact of tweaking the PI3K/Akt signaling pathway known to play an essential role in the regulation of cellular processes, leading to diverse physiological responses. We explored the effects in young (YA, 3–4 months of age) and mature (MA, 11–14 months of age) male and female PDK1 K465E knock-in mice in a battery of tests under different anxiogenic conditions. The results evidenced that the double mutation of the PDK1 pleckstrin homology (PH) domain resulted in an enhancement of the negative valence system shown as an increase of responses of fear- and anxiety-like behaviors in anxiogenic situations. Interestingly, this seemed to be specific of YA and found regulated at middle age. In contrast, cognitive deficits, as measured in a spatial working memory task, were found in both YA and MA mutants and independently of the level of their anxious-like profiles. These distinct age- and function-dependent impacts would be in agreement with the distinct cortical and limbic deficits in the Akt signaling in the brain we have recently described in these same animals. The elicitation of age- and neuronal-dependent specific patterns suggests that fine-tuning the intensity of the PKB/Akt signal that enables diverse physiological response has also its *in vivo* translation into the negative valence system and age is a key regulatory factor.

## Introduction

The understanding of the age-dependent expression of psychiatric symptoms such as anxiety still demands important efforts to unveil and scrutinize its biological and environmental basis through the life span. In the last decade, a new consideration of psychopathology is discussed in terms of dysregulation and dysfunction in essential aspects of behavior based on basic neuroscience and behavioral science research (nimh.nih.gov/research/research-funded-by-nimh/rdoc/index.shtml). The negative valence system (NVS; primarily responsible for responses to aversive situations or context, such as fear, anxiety, and loss) and cognitive system are two of the five tentative domains convened by the National Institute of Mental Health (2020) in its Research Domain Criteria (RDoC) matrix. This matrix results from the crossword between behavioral dimensions or functional constructs and seven different levels of analysis: from genes, molecules, cells, and neuronal circuits to physiology, behavior, and self-report (Asher, 2010). In this context, while basic research on signal transduction is providing a refined close examination of the impact of cell membrane receptors and second messengers on cellular biochemistry and physiology, its downstream actions may be more difficult to characterize even more when related to age. For instance, the phosphatidylinositol 3-kinase (PI3K) signaling pathway, which has been widely involved in controlling neuronal development and function (Waite and Eickholt, 2010). This pathway transmits the extracellular signals through the 3-phosphoinositide-dependent protein kinase-1 (PDK1), an enzyme that emerged as the major transducer of PI3K actions. PDK1 activates at least 23 AGC protein kinase family members besides Akt, the most popular effector of the pathway (Mora et al., 2004; Pearce et al., 2010). Its functional role in cellular biochemistry and physiology as well as cancer and metabolism has been largely explored; however, bottom-up approaches to elucidate the impact of dysfunctional PI3K/Akt signaling in behavior or how it differs through the aging process are still scarce.

The behavioral readout resulting from modulation of the different sites of the PDK1 enzyme is currently studied using new engineered animal models targeting key functional protein domains (Bayascas, 2008). Hypomorphic PDK1 mice, with a reduced general activity of PDK1, showed several behavioral differences related to anxiety and exploration in various tests (Leibrock et al., 2013). We previously generated neuronal-specific conditional knock-in mice in which the expression of the PDK1 L155E mutant form was targeted to neuronal tissues by means of a Nestin-Cre-driven system (Tronche et al., 1999). Disrupting the substrate docking site in PDK1 resulted in the altered activation of several AGC kinases, but intact Akt activation (Cordón-Barris et al., 2016). These animals showed altered activation of several AGC kinases, but intact Akt activation. Their phenotype was characterized by a smaller body size and showed sensorimotor problems, exacerbated disruptive behavior, and cognitive deficits. Oppositely, the PDK1-K465E pleckstrin homology (PH) domain knock-in mice (hereinafter PDK1^−/−^) just have a mutation in the PH domain, only affecting the phosphorylation of PKB/Akt isoforms, but leaving intact activation of the other AGC kinase family members (Bayascas et al., 2008). These mice present a smaller body size and insulin resistance, but their behavioral phenotype and its changes through age are still unknown (Bayascas et al., 2008).

Therefore, the present study was aimed to explore the behavioral phenotype of the PDK1^−/−^ PH domain knock-in mice. We recently reported that the deficits in the Akt signaling are pronounced both in the cortex and the hippocampus during young adulthood (3–4 months of age), but tend to be attenuated by middle age (11–14 months of age) in these mutant mice (Yang et al., 2018). In the present study, the behavioral and functional phenotype screening of these animals was analyzed. We used a battery of four unconditional tests differing on the levels of fear and anxiety and where the sequence of behavioral events that are successively developed in an action program (Lát, 1973) involves cognitive function. Spontaneous exploratory behavior, emotionality, bizarre and anxiety-like behaviors, habituation, as well as working memory were evaluated to address the negative valence and cognitive systems involved.

## Materials and Methods

### Analysis of the PDK1 K465E Mutation

Mutation of Lys465 in PDK1, which forms key interactions with the D3 and D4 phosphates of PtdIns(3, 4, 5)P_3_, to a Glu residue abolished the binding of PDK1 to phosphoinositides and localization at the plasma membrane (Komander et al., 2004). Prior to generating a knock-in mutation, the structure of the isolated PDK1(K465E) mutant PH domain was crystallized and analyzed to ensure that this mutation did not disrupt the overall PH domain fold. The mutant protein was produced in bacteria and expressed with yields similar to those for the wild-type PDK1 PH domain. The overall structure of the PDK1(K465E) PH domain was unaffected by the mutation of Lys465, except for changes observed in the side-chain conformations of residues located in the PtdIns(3,4,5)P_3_-binding pocket. The K465E mutation also significantly reduced the positively charged nature of the ligand-binding interface, which accounts for its inability to bind to phosphoinositides (Komander et al., 2004).

### Generation of PDK1^K465E/K465E^ Mice and Genotyping Analysis

The generation and the genotyping of the PDK1^K465E/K465E^ knock-in mice expressing the single-amino acid substitution of lysine 465 to glutamic acid in the PDK1 PH domain were described previously (Bayascas et al., 2008). The mice were subjected to PCR genotyping of the genomic DNA isolated from ear biopsy using primers K465E F (5′-GGG TGA AGC ATG GAA TCT GTG TCT T) and K465E R (5′-GCC AGG ATA CCT AAG AGT ACC TAG AA). PCR amplification resulted in a 196-bp product from the wild-type allele and a 236-bp product from the targeted allele.

### Animals

A total of 62 mice, PDK1^−/−^ (*n* = 42) and PDK1^+/+^ [also referred to as wild type (WT), *n* = 20], including a pool of both sexes (50%) and two maturation ages, YA (3- to 4-month-old young adults, *n* = 19) and MA (11- to 14-month-old mature adults, *n* = 43), were used.

Mice were maintained at the Animal House Facility of the Universitat de Lleida under standard husbandry conditions (housed three to four per cage in 35 cm × 35 cm× 25-cm Macrolon cages, with food and water *ad libitum*, 22 ± 2°C, a 12-h light/dark cycle, and relative humidity of 50–60%). Behavioral assessments and data analysis were performed blind to the experiment, in a counterbalanced manner, in the light cycle, from 09:00 to 13:00 h. All procedures were in accordance with the Spanish legislation on the “Protection of Animals Used for Experimental and Other Scientific Purposes” and the EU Directive (2010/63/UE) on this subject. The study complies with the ARRIVE guidelines developed by the NC3Rs and the aim to reduce the number of animals used.

### Behavioral Assessments

**Figure 1:**
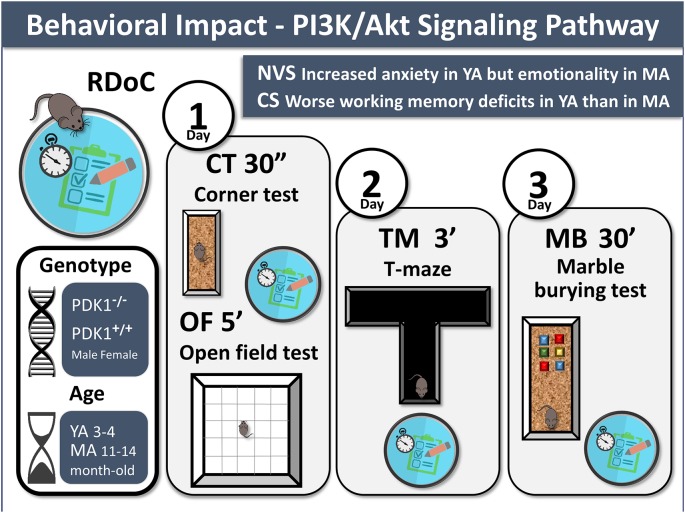
Graphical abstract. Behavioral impact of the PI3K/Akt signaling pathway. Experimental design: 3-days battery of four behavioral tests to assess the Research Domain Criteria (*RDoC*), negative valence system (*NV*), and cognitive system (*CS*) in PDK1^−/−^ and PDK1^+/+^ mice at young age (*YA*) and mature age (*MA*) are illustrated. Main findings are also indicated.

Animals were behaviorally assessed for negative valence and cognitive systems in a battery of tests composed of a corner test, an open field test, T-maze, and marble-burying test. Somatic growth, as measured by body weight, as well as sensorimotor tasks were recorded on day 0 prior to the behavioral battery of tests in order to monitor possible confounding factors. A graphical abstract, also including the conclusions, illustrates the methodological settings and procedures (Figure 1).

#### Day 0: Reflexes and Sensorimotor Tasks

Visual reflex and posterior leg extension reflex were measured three times by holding the animals by the tail and slowly lowering them toward a black surface. Motor coordination and equilibrium were assessed twice (20-s trials) in two consecutive road tasks of increasing difficulty. The distance covered and the latency to fall off a wooden (1.3-cm width) and a metal wire (1-cm diameter) rods (both 1 m long) were recorded.

#### Day 1: Corner Test and Open Field Test

Corner test (CT) was used to evaluate neophobia. The animals were individually placed in the center of a clean standard home cage filled with wood shave bedding and observed for 30 s. We measured the numbers of corners visited (CTc), the latency to realize the first rearing (CTlatR), and the number of rearings (CTr). Once they completed the CT, the mice were placed in the center of an illuminated (20 lx) open field (homemade woodwork, white box, 55 cm × 55 cm × 25 cm) and observed for 5 min. First, the ethogram of action programs (sequence of behavioral events) was analyzed. Thus, the duration of freezing behavior (latM, latency of movement) and the latency of the behavioral events that follow to it were recorded: leaving the central square (latC), reaching the periphery (thigmotaxis, latP), performing first wall rearing (latR), and first grooming (latG). Second, the time course and total levels of exploratory activity were measured as horizontal (C, number of crossings) and vertical (Rw, rearing with wall support) locomotor activity. Third, variables of emotionality included the number of defecations (Def), the presence of urine (Ur), and the grooming behavior, through its number (nG), latency (latG), and its total time (tG). Finally, as previously described (Baeta-Corral and Giménez-Llort, 2014), we evaluated the presence of the following bizarre behaviors: stereotyped rearings without wall support (Rc), recoils (Re), scratching (Sc), turning (T), stretch attendance (Sa), jumping (Ju), and jerks (Jk). Bursting (grooming with a pattern broken in its first step, where the animal is only washing hands) was measured through its number (nB), latency (latB), and total time (tBG).

#### Day 2: T-Maze

Spontaneous alternation of mice was tested in a T-shaped maze (with arms 25 cm in length). The animals were placed inside the “vertical” arm of the maze with the head facing the end wall. The performance was evaluated by determining with a chronometer the time elapsed until the animal crossed (four-paw criteria) the intersection of thee three arms (latT) and the time to complete the test (latF). The task finished when the mice visited the two arms of the maze. The entry of an already visited arm in the trial before completing the test was considered an error (eT).

#### Day 3: Marble-Burying Test

The procedure used was as previously described (Torres-Lista et al., 2015). The mice were placed individually facing the wall in a standard home cage with six glass marbles (1 cm × 1 cm × 1 cm) on a 5-cm-thick layer of clean woodcuttings. The marbles where spaced in three rows of two marbles per row in one half of the cage. The mice were left in the cage with marbles for a 30-min period, after which the test was terminated by removing the mice. The number of marbles buried, changed of position (partially buried or turned), and left intact (I) were measured.

### Statistics

Statistical analyses were performed using SPSS 15.0 software. All data are presented as the mean ± SEM and illustrated as bars (pooled data by genotype or age) or as dots that illustrate the individual values in each group segregated by sex and/or age, as indicated in the legends. To evaluate the effects of genotype (G) and age (A) group, a 2 × 2 factorial analysis design was applied. Differences were studied through multivariate general linear model analysis, followed by *post hoc* Sidak test comparisons, when possible. For categorical variables, the Fisher’s exact test with 2 × 2 and 4 × 2 designs was used. Spearman’s correlation analyzed the body size and behavioral correlates. Graphics were made with GraphPad Prism 6. To explore possible hints of sexual differences, males (squares) and females (circles) were represented with different symbols in the graphics. *P*-value < 0.05 was considered as statistically significant.

## Results

### Somatic Growth/Body Weight

The body weights of the animals (in grams)—YA PDK1^−/−^ mice, 18.67 ± 1.29; MA PDK1^−/−^ mice, 22.90 ± 0.84; YA PDK1^+/+^ mice, 28.14 ± 1.92; YA PDK1^+/+^ mice, 32.00 ± 2.47—showed genotype and age effects (G: *F*_(1,58)_ = 30.692, *P* = 0.000; A: *F*_(1,58)_ = 5.822, *P* = 0.019), with lower body weight of PDK1^−/−^ mice as compared to the age-matched WT and also for younger animals compared to mature animals. *Post hoc* comparisons showed a higher body weight in WT mice as compared to PDK1^−/−^ in both ages (*P* = 0.001 and *P* = 0.000 for YA and MA, respectively). Only PDK1^−/−^ MA exhibited a heavier body weight than their genotype-matched YA counterparts (*P* = 0.036).

### Reflexes and Sensorimotor Tasks

No statistical differences of any type where found in the visual task. All the mice obtained the maximum score possible. In the motor tasks, no genotype differences were found in any of them. Only, an age effect was observed in the second of two trials of the metal rod, with YA mice being able to maintain the equilibrium in the road during more time than did the MA counterparts, as expected for their age-related lower weight.

### Corner Test

**Figure 2:**
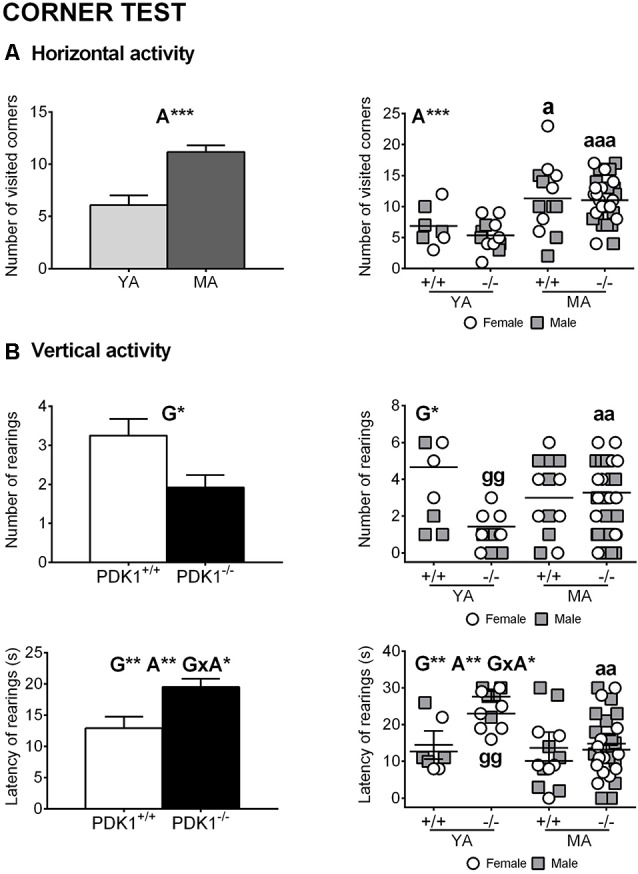
Exploratory behaviors in the corner test in young adult (*YA*) and mature age (*MA*) PDK1^−/−^ and age-matched wild-type (*WT*, PDK1^+/+^) mice. Data are expressed as mean ± SEM. *Bars* illustrate the age or the genotype groups, as indicated in the *abscissae*. *Symbols* are used to illustrate individual values of females (*white circles*) and males (*gray squares*) of each genotype and age group (*YA* and *MA*), as indicated in the *abscissae*. Factorial analysis: *G*, genotype effect; *A*, age effect; *G×A*, genotype × age interaction effects, *P < 0.05, **P < 0.01, ***P < 0.001. *Post hoc* test: genotype (*g*), ^gg^*P* < 0.01 vs. the corresponding WT (PDK1^+/+^) group; age (*a*), ^a^*P* < 0.05, ^aa^*P* < 0.01, ^aaa^*P* < 0.001 vs. the corresponding YA of the same genotype.

Figures 2A,B illustrate the horizontal and vertical exploratory behaviors in the test for neophobia. The 2 × 2 GLM analysis showed genotype effects in the vertical activity, with increased latency of first rearing (G: *F*_(1,58)_ = 8.350, *P* = 0.005) and consequent reduction in the number of rearings (G: *F*_(1,58)_ = 6.367, *P* = 0.014) in the PDK1^−/−^ mice vs. the WT (Figure 2B). Strong age effects also appeared, with higher number of corners visited (A: *F*_(1,58)_ = 20.719, *P* = 0.000) and higher latency of first rearing (A: *F*_(1,58)_ = 7.481, *P* = 0.008) in the MA vs. the YA group. Besides, a genotype × age interaction effect was found in the latency of rearing (G × A: *F*_(1,58)_ = 4.072, *P* = 0.048) since the effect was mostly due to an increase in the PDK1^−/−^ YA group.

The *post hoc* comparisons analysis of the number of visited corners indicated that the performances of both YA PDK1^−/−^ (*P* = 0.000) and YA PDK1^+/+^ (*P* = 0.017) were lower than those of their respective older age groups. Also, YA PDK1^−/−^ mice showed lower number of rearings than their older MA PDK1^−/−^ group (*P* = 0.009) and as compared to the age-matched WT mice (*P* = 0.009) due to an increased latency (*P* = 0.000 and *P* = 0.004, respectively).

### Open Field Test

**Figure 3:**
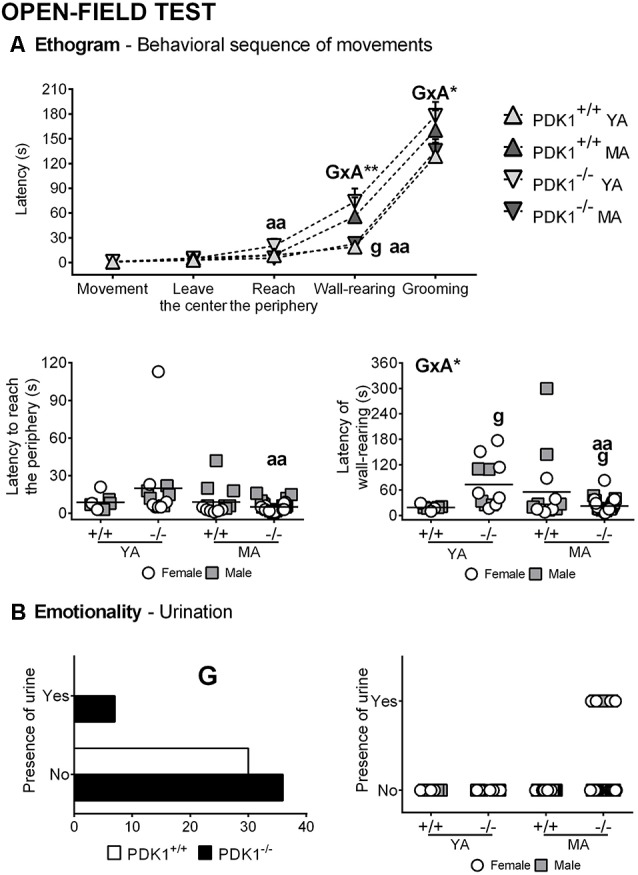
Ethogram and emotionality in the open field test in young adult (*YA*) and mature age (*MA*) PDK1^−/−^ and age-matched wild-type (*WT*; PDK1^+/+^) mice. Data are expressed as the mean ± SEM or incidence. *Bars* illustrate the genotype groups, as indicated in the *Y*-axis. *Symbols* are used to illustrate the different groups or individual values, as depicted in the *legends* or *abscissae*. Factorial analysis: *G*, genotype effect; *A*, age effect, *P < 0.05, **P < 0.01. *Post hoc* test: genotype (*g*), ^g^*P* < 0.05 vs. the corresponding WT (PDK1^+/+^) group; age (*a*), ^aa^*P* < 0.01 vs. the corresponding YA of the same genotype.

**Figure 4:**
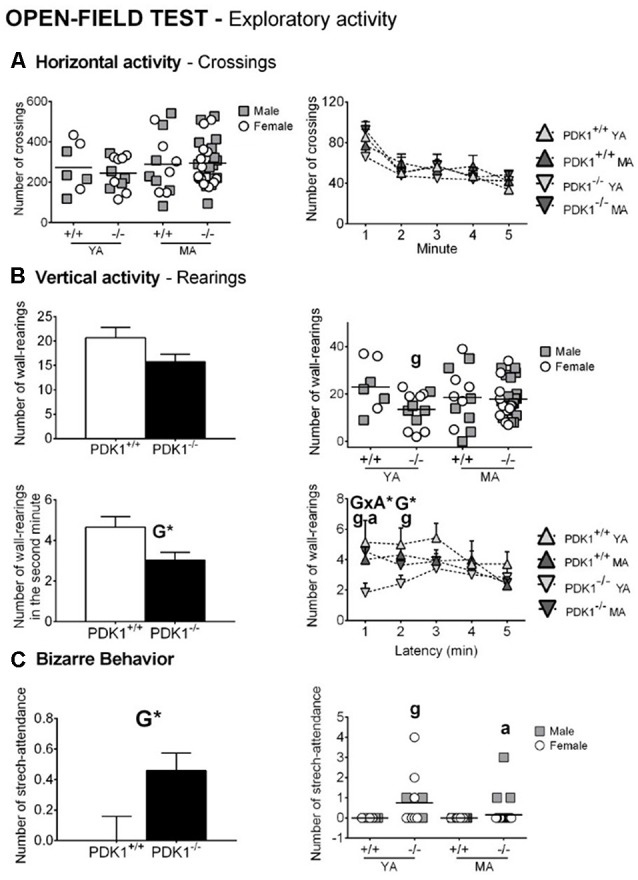
Exploratory and bizarre behaviors in the open field test in young adult (*YA*) and mature age (*MA*) PDK1^−/−^ and age-matched wild-type (*WT*; PDK1^+/+^) mice. Data are expressed as the mean ± SEM. *Bars* illustrate the genotype group, as indicated in the *abscissae*. *Symbols* are used to illustrate the different groups or individual values, as depicted in the *legends* or *abscissae*. Factorial analysis: *G*, genotype effect, *P < 0.05. *Post hoc* test: genotype (*g*), ^g^*P* < 0.05 vs. the corresponding WT (PDK1^+/+^) group; age (*a*), ^a^*P* < 0.05 vs. the corresponding YA of the same genotype.

Figures 3, 4 depict the main behavioral domains, events, and units of analysis in the open field test, showing a distinct performance on PDK1^−/−^ compared to the WT groups. Although no differences were found in the latency of first movement or latency to leave the center (Figure 3A), bizarre behaviors (Figure 4C), scarcely elicited in the WT, were conspicuously observed in the PDK1^−/−^ mice. Thus, an increased number of stretch attendance was found in the PDK1^−/−^ genotype (G: *F*_(1,58)_ = 5.460, *P* = 0.023; Figure 4C). A genotype main effect was found in the number of wall rearings in the second minute (G: *F*_(1,58)_ = 6.126, *P* = 0.016; Figure 4B), with a decreased number for the PDK1^−/−^ mice. Genotype × age interaction effects were shown in the latency (G × A: *F*_(1,58)_ = 10.796, *P* = 0.002; Figure 3A) and number of wall rearings in the first minute of the test (*F*_(1,58)_ = 4.595, *P* = 0.036; Figure 4B), as well as in the latency of grooming (*F*_(1,58)_ = 4.139, *P* = 0.046; Figure 3A).

The *post hoc* comparisons analysis showed that the ethogram and variables of analysis of YA PDK1^−/−^ mice were indicative of a worse performance as compared to their older MA PDK1^−/−^ counterparts. Thus, YA PDK1^−/−^ exhibited more stretch attendances (*P* = 0.014), reached the periphery later (*P* = 0.004), exhibited longer latency for wall rearing (*P* = 0.002), and a consequent low number of this behavior in the first minute of the test (*P* = 0.013). As compared to the age-matched WT animals, the stretch attendance of YA PDK1^−/−^ was also higher (*P* = 0.023) and the latency of wall rearing was delayed (*P* = 0.016), resulting in a lower number of rearing during the first (*P* = 0.028) and the second minute (*P* = 0.020) of the test and, consequently, a lower total number of rearings (*P* = 0.031). At mature ages, MA PDK1^−/−^ exhibited faster apparition of rearing (*P* = 0.034) as compared to their age-matched WT counterparts. Finally, emotionality, as measured by the presence or not of urine and defecations, was analyzed by Fisher’s exact test with 2 × 2 and 4 × 2 designs. Only for the urination were the two designs significant (*P* = 0.01458 and *P* = 0.032, respectively). The detailed representation per group hints that the enhanced urination in PDK1^−/−^ mice was mostly due to MA PDK1^−/−^ mice.

### Spontaneous Alternation in the T-Maze

**Figure 5:**
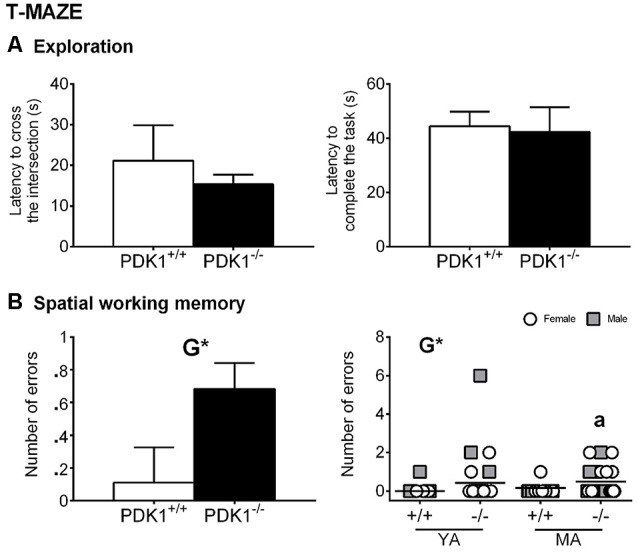
Spontaneous alternation in the T-maze in young adult (*YA*) and mature age (*MA*) PDK1^−/−^ and age-matched wild-type (*WT*; PDK1^+/+^) mice. Data are expressed as the mean ± SEM. *Symbols* are used to illustrate individual values of females (*white circles*) and males (*gray squares*) for each genotype and age group. Factorial analysis: *G*, genotype effect, *P < 0.05. *Post hoc* test: age (*a*), ^a^*P* < 0.05 vs. the corresponding YA of the same genotype.

Although the latency to cross the intersection of thee three arms and the latency to complete the task were similar among all groups, a trend of reduced exploratory activity was shown by PDK1^−/−^ mice (Figure 5A). A genotype effect was found in the number of errors, with PDK1^−/−^ mice exploring more visited arms (G: *F*_(1,58)_ = 4.652, *P* = 0.035; Figure 5B) than did the WT mice, where the incidence of errors was *n* = 1 for each age group. The *post hoc* comparisons analysis also showed an increased number of errors in the YA PDK1^−/−^ as compared to its older genetic counterparts (*P* = 0.048; Figure 5B, right).

### Marble Burying Test

**Figure 6:**
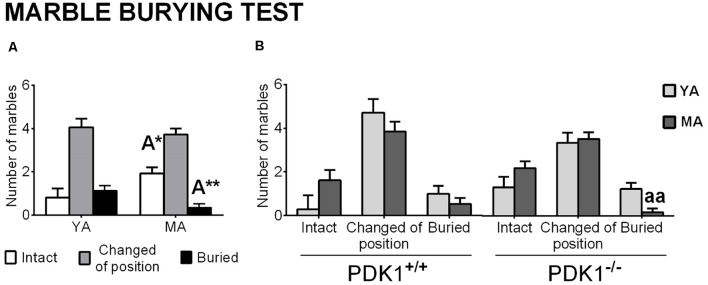
Marble burying in young adult (*YA*) and mature age (*MA*) PDK1^−/−^ and age-matched wild-type (*WT*; PDK1^+/+^) mice. Data are expressed as the mean ± SEM. *Bars* are used to illustrate the different levels of marble burying, as depicted in the *abscissae*. +/+, PDK1^+/+^ genotype group; −/−, PDK1^−/−^ genotype group. Factorial analysis: *A*, age effect, *P < 0.05, **P < 0.01. *Post hoc* test: age (*a*), ^aa^*P* < 0.01, vs. the corresponding YA of the same genotype.

Analysis of the interaction with marbles (Figure 6) pointed at age effects, with a reduced number of marbles buried (A: *F*_(1,58)_ = 7.481, *P* = 0.009) and an increase in those left intact (A: *F*_(1,58)_ = 4.967, *P* = 0.030) in the MA groups as compared to YA mice (Figure 6A). The *post hoc* comparisons analysis revealed that this age pattern was more evident in the PDK1^−/−^ genotype, where MA PDK1^−/−^ mice scarcely buried marbles as compared to their younger genetic counterparts (*P* = 0.002; Figure 6B).

### Body Weight and Behavioral Correlations

**Figure 7:**
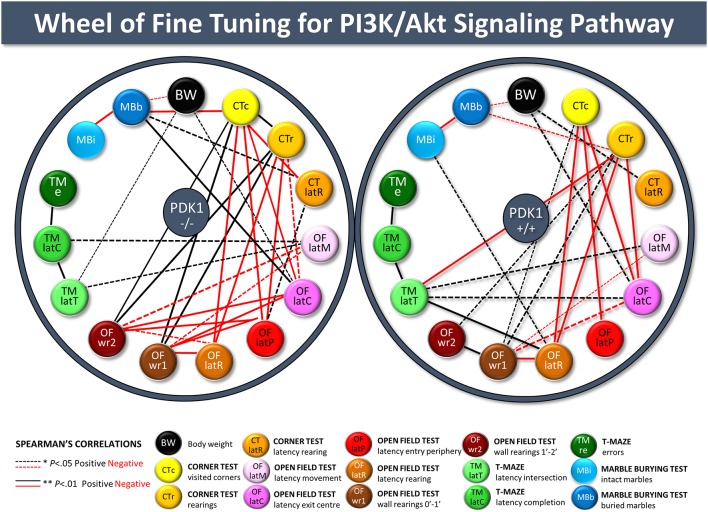
Wheels of fine-tuning for the PI3K/Akt signaling pathway. Illustration of the meaningful behavioral correlates among variables of the different tests in the whole sample of animals, PDK1^−/−^ and age-matched wild-type (*WT*; PDK1^+/+^) mice. Spearman’s correlation analyzed the relationship between behavioral variables of the four behavioral tests and with somatic growth (body weight). *P value < 0.05 was taken as statistically significant. Positive (*black*) and negative (*red*) statistically significant correlations are plotted, with *thickness of the dotted* (*P < 0.05) and *unbroken* (**P < 0.01) *lines* being proportional to the statistically significant value of the correlation.

Spearman’s correlations between and within-test variables are detailed in Supplementary Table S1 and summarized as follows. The body weight of the WT was correlated with the latency of rearing in the CT (*r* = 0.558, *P* = 0.011), while in the PDK1^−/−^ genotype and the total sample of mice, weight was negatively correlated with the number of marbles buried (*r* = −0.312, *P* = 0.044 and *r* = −0.326, *P* = 0.01, respectively). A part of the inter-test correlations between the different variables of a test, behavioral variables of the corner and the open field test were highly correlated and confirmed the results. Neophobia in the corner test strongly predicted the subsequent behavioral ethogram (negative correlation with latencies: all *r* < −0.345, *P* = 0.006), the vertical activity in the open field test (OF; positive correlations with wall rearing in the first 2 min of the test), and the performance in the marble test (*r* = −0.326, *P* = 0.01). Similarly, neophobia in the corner and open field tests (ethogram) was correlated with the ethogram of behavioral events in the T-maze (all *r* > 0.253, *P* = 0.048). The number of errors in the T-maze was correlated with the time invested to explore the test (*r* = 0.528, *P* = 0.000) and the latency of rearing in the corner test (*r* = 0.253, *P* = 0.048). Finally, we propose an illustration of meaningful correlations as wheels (see Figure 7), where distinct fine-tuning of PKI3 signaling in PDK1^−/−^ and WT can be visually observed in an easier manner.

The data that support the findings of this study are available from the corresponding author upon reasonable request.

## Discussion

Brain-based evidence on the impact of dysregulation and dysfunction, from basic neuroscience to behavioral levels, is leading to a new conceptualization of mental health and disease. Thus, the strategic plan of NIMH is “to develop new ways of classified disorders based on dimensions of observable behaviors and brain functions.” Far from due to discrete etiologies, the RDoC Project considers phenotypic differences observed among disorders as explained by variations in the nature and degree of neural circuitry disruptions, under the modulation of several developmental, compensatory, environmental, and epigenetic factors (Hyman, 2007; Morris and Cuthbert, 2012). Here, we can now also add “and age/aging factors.” Thus, dysfunction and dysregulation at the genetic, neural, and behavioral levels point at the fine-tuning of broadly spread networks as critical for a wide array of behaviors and mental processes (Morris and Cuthbert, 2012). In this context, tweaking of the PI3K/PDK1/Akt signaling pathway in the PDK1 K65E knock-in mice unveiled thresholds for its essential role in the regulation of cellular processes, leading to diverse physiological responses (Zhou et al., 2014).

PI3K plays fundamental roles in regulating virtually every physiological process related to cell growth, proliferation, survival, and metabolism, where Akt was meant for decades to represent the major, and perhaps unique, effector mediating these cellular responses. However, since it was first identified as the apical Akt kinase, PDK1 emerged as a major transducer of PI3K actions by regulating a number of AGC kinase family members besides Akt, including S6K, RSK, SGK, and PKC isoforms (Mora et al., 2004). The essentiality of this enzyme has been widely demonstrated by the severe phenotypes reported in different PDK1-deficient mice models (reviewed in Bayascas, 2010). Thus, the PDK1/Akt pathway has been reported as intermediating in the role of neurotropic factors, like brain-derived neurotrophic factor (BDNF), in the acquisition of fear and mood disorders like depression and anxiety (Chen et al., 2006; Duman and Monteggia, 2006; Ou and Gean, 2006; Martinowich et al., 2007). The reduced general activity of PDK1 exhibited by the hypomorphic PDK1 mice (PDK1hm) results in several behavioral differences related to anxiety and exploration assessed in various tests (Ackermann et al., 2008), while cognitively unimpaired Akt2 knockout mice (Akt2^−/−^) presented an anxiety- and depressive-like phenotype (Leibrock et al., 2013). More recently, the increase of Akt phosphorylation has been implicated in the rapid antidepressant-like effects of different drugs in the basolateral amygdala, hippocampus, and the prefrontal cortex (Shi et al., 2012; Wang et al., 2015; Tao et al., 2016). Conversely, mice with high anxiety-related behaviors showed a stronger acquisition, slower extinction, and spontaneous recovery of learned fear that coincide with enhanced phosphorylation of Akt in the amygdala (Yen et al., 2012).

Biochemical and structural data permitted the rational design of two new PDK1 single-amino acid mutations within either the PH domain, namely PDK1 K465E, or the PIF-pocket domain, termed L155E, impeding the activation of Akt or substrates other than Akt, respectively. Phenotypic characterization of two knock-in mouse strains expressing each of these mutations allowed narrowing down the contribution of Akt compared to the complementary PDK1 branch in mediating PI3K actions (Bayascas et al., 2008). The PDK1 K465E mice with reduced activations were viable and exhibited no adverse phenotypes other than being smaller (Bayascas et al., 2008; Zurashvili et al., 2013). In contrast, the PDK1 L155E mice were embryonically lethal, and Cre-mediated bypass of the lethal period by targeting the expression of the mutant PDK1 L55E protein to the brain led to neurodevelopmental disorders and disrupted behavior, thereby highlighting the importance of the Akt-independent actions of this signaling toolkit regarding brain development and function (Bayascas et al., 2008). Nevertheless, in spite of the absence of overt phenotypes, the behavioral consequences of PDK1 K465E knock-in mutation and Akt activity ablation were never explored.

The present work describes, for the first time, the *in vivo* effects of PDK1 mutation in the PH domain. We studied a set of male and female PDK1^−/−^ PH-domain transgenic (PDK1^−/−^) mice at two stages of adult maturation—young adulthood and middle age—and as compared to age-matched WT mice with normal aging. We have recently shown, at the biochemical level, that these animals exhibited pronounced deficits in Akt signaling both in the cortex and the hippocampus during young adulthood, but tend to be attenuated at middle age (Yang et al., 2018). Here, behavioral and functional screening for negative valence systems and cognitive systems indicated that the deregulation of this signaling pathway also has a higher impact at young adulthood, as shown by their increased anxiety-like behavioral profile and working memory deficits, while changes in middle-aged animals were found restricted to emotionality. Somatic growth, as measured by body weight, was reduced, as previously described (Bayascas et al., 2008; Zurashvili et al., 2013).

The corner test for mild neophobia to a familiar environment, such as a standard home cage with new bedding, showed delayed and reduced number of rearings in the mutant mice (mostly in the YA animals), leading to genotype differences and age interaction effects. The correlation analysis showed that both horizontal and vertical activities in the corner test were intercorrelated, mostly in the PDK1^−/−^ genotype. In agreement, a similar but smoother pattern was shown in the horizontal exploratory behavior of YA PDK1^−/−^, albeit it did not reach statistical significance. Differences in the horizontal activity due to the age factor were notorious, indicating lower neophobia response in middle-aged animals, independently of the genotype. This could be explained by long-term habituation to husbandry routines, reducing the chances of animals to perceive a new home cage as a potential threat.

The results of increased neophobia in the corner test were confirmed in a more anxiogenic test such as the open and illuminated field. There, YA mutants showed a change in thigmotaxis, with a delay to reach the periphery and delayed latency for wall rearings. These changes in the behavioral sequence of events of the ethogram were due to the conspicuous appearance of stereotyped stretch attendance and stereotyped rearing in mutant mice, independently of age. These risk assessment behaviors in the open field were scarcely exhibited by 6-month-old wild-type C57BL/6 × 129Sv mice, but were found enhanced in animal models with anxious-like profiles (Komander et al., 2004; Baeta-Corral and Giménez-Llort, 2014). Their presence in those mutants, considered as bizarre behaviors triggered by the extereoceptive anxiogenic stimuli of the open and illuminated arena, was related to high levels of anxiety and was reversed by early neonatal handling (Baeta-Corral and Giménez-Llort, 2014). In the case of PDK1^fl/fl^ CRE^+^ mice, middle-aged females also exhibited stereotyped stretching as part of a more severe disruptive behavior that included vocalizations and hyperactivity and refusal to be handled and tested (Cordón-Barris et al., 2016). Enhanced risk assessment or vigilance is part of the pattern of responses to potential harm (anxiety), included in the behavioral dimensions or constructs within the NVS domain together with responses to acute threat (fear) and to sustained threat, frustrative non-reward, and loss (nimh.nih.gov). PDK1^−/−^ mutation enhanced the anxious responses elicited in a potentially harmful environment. Among the variables of emotionality, the latency of grooming was normal, but urination was present in the PDK1^−/−^ genotype due to incidence in the middle-aged mutants.

A striking parallelism in the ethogram was found in middle-aged mutants and young adult WT, both in the temporal sequence of behavioral events and the level of their expression. The action programs described by Lát (1973), which are consecutively developed by animals when confronting a new environment, were not distinguishable of those found at young adulthood, except for the first minute/s of the test when the first action, which is related to fear, was elicited. Again, the freezing behavior of YA mutant mice evidenced an increased neophobic response, here expressed in terms of a 50% reduction of rearings during the first 2 min of the test. Freezing behavior and reduced total rearings were also shown by middle-aged female PDK1^fl/fl^ CRE^+^ mice, although an overall drop of activity did not allow detecting differences in thigmotaxis (Cordón-Barris et al., 2016).

In the spontaneous alternation task in the black T-maze resembling natural burrows, an innate alternation form of a win–shift behavior of foraging in the wild was elicited. In most of the WT mice, alternation behavior was performed without errors or only once in a couple of cases. In contrast, at both ages, most mutant mice explored already visited areas of the maze, which were considered errors attributed to working memory problems and prefrontal cortex dysfunction, characteristic of schizophrenia-like patterns (Goldman-Rakic, 1994). Alternation behavior has been shown to reflect short-term habituation of responding to stimuli based on their relative familiarity because of recent exposure (Sanderson and Bannerman, 2012). Importantly, similarly to the rewarded alternation and win–shift behavior on the radial arm maze, spontaneous alternation is sensitive to hippocampal lesions (Deacon et al., 2002). The role of the prefrontal cortex is under discussion and considered by these authors (Sanderson and Bannerman, 2012) as being rather involved in other more complex and demanding goal-directed behaviors where active maintenance of information is required (Miller and Cohen, 2001). Here, again, the worse performance was mostly observable in the YA mutant mice, whose brains exhibited more pronounced deficits in Akt signaling in both the cortex and the hippocampus (Yang et al., 2018), key neuroanatomical areas for these behaviors. These cognitive deficits are interesting to note since the mutations related to the PDK1 and Akt signaling pathway, such as those in Akt2 knockout mice, presented a higher anxiety-like and depressive-like behavior, but were cognitively intact (Ackermann et al., 2008). As mentioned above, middle-aged female PDK1^fl/fl^ CRE^+^ mice also presented smaller size as well as sensorimotor problems, exacerbated bizarre behavior, and short-term memory deficits in the Morris water maze (Cordón-Barris et al., 2016). Therefore, while the anxious-like patterns seem to be more sensitive to be affected, a tiny modulation and Akt signal thresholds may determine distinct levels of cognitive system dysfunction.

Similarly to the elevated T-maze (Asth et al., 2012), the T-maze can be used as an animal model to simultaneously investigate memory and anxiety in mice. Thus, the latency to reach the intersection of the arms is considered a measure of copying with stress strategy, and it is used to measure anxiety in very mild conditions (Guayerbas et al., 2000). In this respect, correlations with the variables related to neophobia in the corner and open field tests were found when the whole sample was analyzed and in each genotype. However, no differences were found between genotypes, but a trend of shorter latency to reach the intersection was shown by mutants, albeit it did not reach statistical significance.

Finally, mice were assessed in the marble burying test, which has been successfully used for the pharmacological assessment of obsessive–compulsive behaviors, anxiety, and psychosis drugs (Jimenez-Gomez et al., 2011; de Brouwer et al., 2019). The pattern, with predominance in the incidence of “changed or partially buried” marbles and equal low numbers of marbles buried and left intact, shown by all YA animals, resembled that already reported by our laboratory in middle-aged males of the standard hybrid C57BL/6 × 129sv strain (Torres-Lista et al., 2015). Here, independently of the genotype, the MA animals showed a shift to the left, with increased numbers of marbles left intact in detriment of those buried as compared to the burying pattern shown by YA animals. This age effect was more clearly expressed in the PDK1^−/−^ genotype, with the reduction of burying in MA *vs*. YA mice reaching statistical significance. This drop in behavior resembled the effect obtained in wild-type mice after a chronic low 1-mg/kg, s.c, non-cataleptic dose of risperidone (Bardin et al., 2006; Baeta-Corral and Giménez-Llort, 2014). A similar trend was shown in middle-aged female PDK1^fl/fl^ CRE^+^ mice, whose number of buried marbles was in detriment in favor of a statistically significant increase in the number of marbles only changed of position or half buried as compared to their age-matched wild-type counterparts (Cordón-Barris et al., 2016). With respect to the integration of the results of the marble test with the other tests, meaningful correlations were found, with the number of buried marbles being directly related to neophobia in the corner test in both WT (negative correlation with the number of rearings in the corner test) and the PDK^−/−^ (negative correlation with the number of visited corners) genotypes. This was in agreement with our previous results in middle-aged standard hybrid C57BL/6 × 129sv (Baeta-Corral and Giménez-Llort, 2014), where the number of buried marbles correlated with the latency of rearing in the corner test and the incidence of urine, the variable that was found increased in MA PDK^−/−^ mice.

In summary, the double mutation of the PDK1 PH domain resulted in an enhancement of NVS, shown as an increase of responses of fear- and anxiety-like behaviors in anxiogenic situations, which seemed to be specific of young adulthood and found regulated at middle age, where only an increased emotionality was noted. In contrast, cognitive deficits, as measured in a spatial working memory task, were found in both YA and MA PDK^−/−^ mice and independently of the level of their anxious-like profiles. This would be in agreement with the distinct cortical and hippocampal deficits in the Akt signaling in the brain of these animals (Bayascas et al., 2008). The present results contribute to the *in vivo* characterization of the behavioral impact of Akt signaling pathway dysfunction and support the conceptualization of qualitative and quantitative variations of neural circuit disruptions underpinning phenotypic differences that are relevant to several mental disorders. The elicitation of age-dependent specific patterns suggests that fine-tuning the intensity of the Akt signal enables not only diverse physiological responses, as we have previously demonstrated in these animals, but also their readout *in vivo*. Finally, the modulation by sex factor will deserve further exploration.

## Data Availability Statement

The raw data supporting the conclusions of this article will be made available by the authors, without undue reservation, to any qualified researcher.

## Ethics Statement

The animal study was reviewed and approved by CEEAH.

## Author Contributions

LG-L designed and performed the behavioral tests and illustrated the graphical abstract and the proposed wheels of correlation. MS-S constructed and analyzed the matrix of data and illustrated the results. JB provided the animals and financial support. All authors participated in the scientific discussions and contributed to the writing of the manuscript.

## Conflict of Interest

The authors declare that the research was conducted in the absence of any commercial or financial relationships that could be construed as a potential conflict of interest.

## Supplementary Material

TABLE S1 Behavioral correlates among variables of the different test in the wholes sample of animals, PDK1^−/−^ and age-matched WT (PDK1^+/+^) mice. Spearman’s correlation analyzed the relationship between behavioral variables of the four behavioral tests and with somatic growth (body weight). **P*-value < 0.05 was taken as statistically significant; ***P*-value < 0.01.
